# Synthesis and Characterization of Poly(2-vinylpyridine) and Poly(4-vinylpyridine) with Metal Oxide (TiO_2_, ZnO) Films for the Photocatalytic Degradation of Methyl Orange and Benzoic Acid

**DOI:** 10.3390/polym14214666

**Published:** 2022-11-01

**Authors:** Iririana Martínez, Ricardo Santillán, Iliana Fuentes Camargo, Julia Liliana Rodríguez, J. Alberto Andraca Adame, Hugo Martínez Gutiérrez

**Affiliations:** 1 Laboratorio de Investigación en Ingeniería Química Ambiental, ESIQIE—Instituto Politécnico Nacional, Zacatenco, Ciudad de México 07738, Mexico; 2 Laboratorio de Polímeros, ESIQIE-Instituto Politécnico Nacional, Zacatenco, México, Ciudad de México 07738, Mexico; 3 Departamento Ciencias Básicas, UPIIH—Instituto Politécnico Nacional, Mexico City 42050, Mexico; 4 Centro de Nanociencias y Micro y Nanotecnologías, Instituto Politécnico Nacional, Ciudad de México 07738, Mexico

**Keywords:** 4-vinylpyridine, 2-vinylpyridine, photocatalyst, TiO_2_, ZnO, polymer, film

## Abstract

In this study, composite material films of pyridine-based polymer and metal oxides (ZnO and TiO_2_) were successfully deposited by spin coating method for environmental remediation. Firstly, the polymers poly(2-vinylpyridine) P(2-VP), and poly(4-vinylpyridine) P(4-VP) were synthesized via solution polymerization. The analysis by grazing incidence X-ray diffraction (GIXRD) reveals semicrystalline nature and scanning electron microscopy (SEM) indicates that the poly(vinylpyridines) clusters of particles were observed on the surface of the films. It was also shown that the morphology of composite materials is completely dependent on the chemical nature of the oxide. In the case of P(2-VP)-TiO_2_ and P(4-VP)-TiO_2_, some channels or pathways of TiO_2_ on the surface of films were observed. However, the surface morphology of the polymer composites formulated with ZnO shows a homogeneous distribution in P(2-VP) and P(4-VP) matrix. The effectiveness of the composite materials in the photodegradation of methyl orange (MO) was evaluated by photocatalysis. According to the results, the P(4-VP)-ZnO composite exhibited the highest photodegradation of MO, allowing the separation of photogenerated species required for the photocatalytic reaction. The P(4-VP)-ZnO composite was also tested in benzoic acid (BA) photodegradation in water. The presence of some scavengers in the reaction system reveals that hydroxyl radicals (OH•), superoxide radicals (O_2_-•) and holes (h+) are responsible for the BA reduction by photocatalysis.

## 1. Introduction

The wide diversity of organic compounds and toxic heavy metals present in water bodies has led to the severe problem of pollution that affects aquatic as well as human life [1,2,3]. Generally, some of these pollutants come from textile [4,5], petrochemical [6], paper [7], and pharma industries [8,9], among other human activities due to the discharge of untreated waste matter. Traditional methods are not enough for the treatment of those effluents because the organic pollutants are recalcitrant and non-biodegradable. Hence, advanced oxidation processes (AOPs) are considered effective and sustainable [10,11]. The main advantage of AOPs is the in situ production of highly oxidant species, such as superoxide anion radicals (O_2_•^−^), hydroxyl radicals (•OH), and sulfate radicals (SO_4_•^−^), which achieve the mineralization of refractory organic pollutants [12,13]. Among AOPs, the photocatalysis treatment has attracted a lot of attention for degrading the toxic organic pollutants from water [14,15]. 

Usually, the photocatalysis process utilizes a material that absorbs light irradiation (UV or visible) for subsequent photogeneration of reactive species (electrons and holes), which migrate to active sites and initiate redox reactions. O_2_•^−^ radicals are formed by the reaction between the oxygen with free electrons while •OH radicals are due to energized holes that react with hydroxyl anions or water molecules.

Generally, inorganic photocatalysts such as metal oxide, sulfides and nitrites are used as semiconductors (SCs) for environmental remediation [16,17]. It is well known that titanium dioxide (TiO_2_) is the most studied photocatalyst, and the second is undoubtedly zinc oxide (ZnO) [18,19]. Both semiconductors are low-cost and environmentally friendly and their energy levels are located almost at the same positions [20,21]. Nevertheless, their wide bandgap energy limits the absorption of solar light and causes the fast recombination of photogenerated species. However, TiO_2_ and ZnO are highly stable in colloidal suspension, therefore their separation and recovery after water treatment are complicated [22]. Many studies have been carried out to improve their photocatalytic activity; for instance, the incorporation of conductive metals (plasmonic structures, metal doping), graphene coating, heterojunction formation of two or more semiconductors, among others [23,24,25]. 

An alternative to reduce the recombination of photogenerated electron–hole pairs in semiconductors is the incorporation of conductive polymers [26]. Polymers provide matched band structures with inorganic photocatalyst in the composite and produce a strong interfacial effect between conjugated materials [27]. This outcome promotes charge separation and reduces the charge recombination rate during the electron transference [28]. The common types of conductive polymers are polyaniline (PANI) [29], poly(3,4-ethylenedioxythiophene) (PEDOT) [30], polypyrrole (PPy) [31], polythiophene (PTh) [32], and their derivate. The most used hybrid composite (conductive polymer–metal oxide) is PANI with TiO_2_ which can photodegrade several pesticides, dyes, and pharmaceuticals under the irradiation of UV or visible light [33,34]. Nevertheless, PANI is synthesized by chemical oxidative polymerization under strongly acidic conditions at sub-zero temperatures [35]. It is important to consider the degree of oxidation of the nitrogen atoms, and PANI can be classified in three forms: fully reduced, half or fully oxidized [36]. The first form of PANI is considered an insulator, therefore it is necessary to regulate the oxidation degree. 

Nowadays, polyvinylpyridine (PVP) is a good polymer to enhance the electrocatalytic activity and the electron transfer kinetic in sensors in electrochemical applications [37]. Generally, PVP has been used for the removal of anionic compounds hence its pyridine ring can be quaternized to promote ion exchange [38,39]. Another application of PVP is as sorbent for the removal of organic compounds from wastewater. Chanda et al. [40] reported an excellent capacity of the cross-linked poly(4-vinylpyridine) polymer to adsorb phenolic species (p-aminophenol, p-cresol, p-chlorophenol, and p-nitrophenol) from aqueous solution. PVP has a unique chemical structure and due to its chemical characteristics, it has also been applied as a photocatalyst in the degradation of pollutants. Liu et al. [41] synthesized a polymer–crystalline TiO_2_ nanocomposite through copolymerization of divinylbenzene with 1-vinylimidazolate or 4-vinylpyridene. Both nanocomposites showed excellent activity and good recyclability in the photodegradation of p-nitrophenol and rhodamine-B in water under visible light irradiation. Therefore, in the current work, a novel methodology is presented for the preparation of composite films with good photocatalytic activity to mitigate environmental pollution. P(2-VP) and P(4-VP) were prepared via solution polymerization using two vinyl groups of the monomer 2-vinylpyridine (2-VP) and 4-vinylpyridine (4-VP), respectively. P(2-VP)-TiO_2_, P(2-VP)-ZnO, P(4-VP)-TiO_2_, and P(4-VP)-ZnO composites were prepared by spin coating method on glass substrate. Several characterization techniques were applied to show the morphologies and physicochemical features of composites. Degradations of methyl orange (MO) dye and benzoic acid (BA) were performed to evaluate the photocatalytic activity of the composites under UV light irradiation (λ = 365 nm). 

## 2. Materials and Methods

### 2.1. Reagents and Materials

TiO_2_ (nanopowder, 21 nm), ZnO (nanopowder, 100 nm), 2-VP, 4-VP, MO, BA, isopropyl alcohol (IPA) as solvent, benzoyl peroxide (BPO) as initiator, and p-benzoquinone and ascorbic acid as scavengers were purchased from Sigma-Aldrich. All reagents were used as received without further purification. 

### 2.2. Synthesis of P(2-VP) and P(4-VP)

The monomer (30 g) was dissolved in IPA (70 g) followed by the addition of the initiator (BPO). In this work were considered two reaction systems (RSs) and, in the first (RS1), the above solution was transferred to a 250 mL round-bottom flask, and it was stirred with a magnetic stirrer for 6 or 24 h, Figure S1. The effect of reaction temperature (55, 65, and 75 °C), initiator:monomer weight ratio (2 or 4 wt%), and monomer type (2-VP, 4-VP) were studied in the RS1. According to experimental conditions obtained from the RS1, the reaction temperature was established for each pyridine, and the initiator:monomer weight ratio and reaction temperature were also fixed in the second reaction system (RS2). In this case, the solution was transferred to a jacketed reactor and stirred with a metal propeller (Caframo, DBC2010) to create mechanical agitation, Figure S2. The monomer conversion was determined from the calculation of the solids percentage.

### 2.3. Preparation of PVP–Metal Oxide Composites

First, 10% wt of P(2-VP) or P(4-VP) solutions were obtained by dilution with IPA from reactor solutions, and then the solution was deposited on a glass substrate by using a spin coating technique at room temperature and, after the deposition, it was dried at 80 °C for 2 min. Subsequently, 2 mL of metal oxide suspension (3 g L^−1^, TiO_2_ or ZnO) was uniformly added to the surface of the polymer. Finally, PVP–metal oxide composites were subjected to a similar thermal treatment as the polymer, Figure S3. All obtained films were immersed in 250 mL water and stirred for 48 h to remove unreacted monomer, followed by drying at room temperature. It is important to mention that the composite films were realized with the synthesized PVPs by RS2 due to the higher conversions of polymerizations that were obtained.

### 2.4. Films Characterization

Grazing incidence X-ray diffraction (GIXRD) patterns were recorded on a PANalytical model Empyriam diffractometer with Cu Kα radiation (45 kV and 40 mA). The incidence grazing angle of the X-ray beam was fixed at 0.5° and 2-theta scanning angle (20° to 80°), the pixcel detector step was 0.01. Morphology studies of polymer and composites films were carried out using optical and scanning electron microscopy (SEM). The microscopic observation was performed in situ with an optical microscope (Premiere, MIS-9000 T, tungsten lamps) with a Moticam camera (1 Mpx). The bright- and dark-field imaging of composites were further obtained by an Olympus BX51 microscope equipped with Luminera camera at magnification from 5X to 20X. SEM investigations were performed with a Jeol-JSM 7800 F microscope. The Fourier transform infrared (FTIR) spectra of the films were recorded on a Perkin Elmer Spectrum 65. The ultraviolet–visible diffuse reflectance spectroscopy (UV–Vis DRS) was performed using a Dynamica (HALO BD-30) double beam spectrophotometer from 200 to 700 nm. 

### 2.5. Degradation Test

The photocatalytic degradation of MO and BA was carried out in a square glass under irradiation by UVA lamp (Tecnolite, 365 nm, 10 W). A typical run consisted of two films placed parallel to the irradiation source into a square glass containing 200 mL of MO or BA (10 mg L^−1^) aqueous solution. Prior to irradiation, the solution was magnetically stirred in the dark for approximately 30 min. At certain time intervals, 3 mL aliquots were withdrawn an analyzed by UV–Vis spectroscopy (Lambda 25, Perkin Elmer) at 230 and 465 nm for BA and MO, respectively. 

## 3. Results and Discussion 

### 3.1. Temperature Effect and Initiator: Monomer Ratio during the Polymerization 

**Figure 1:**
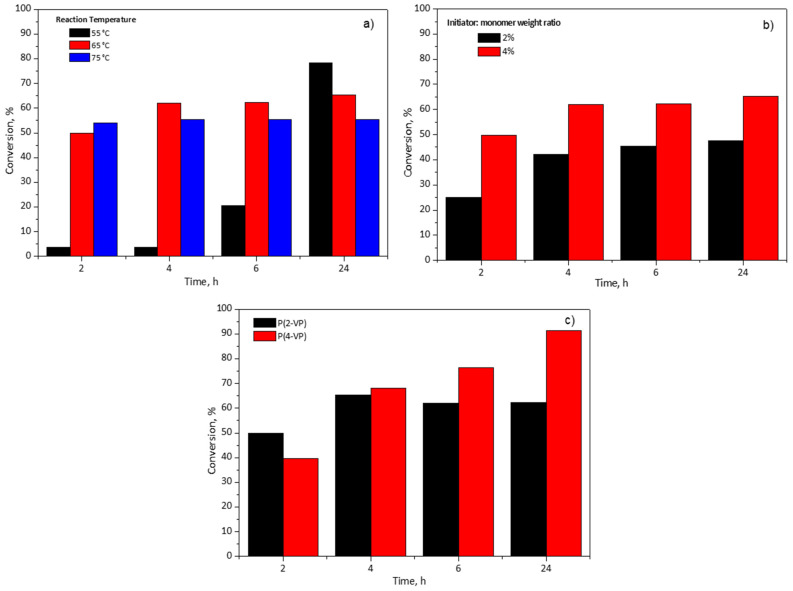
Effect of (**a**) reaction temperature of P(2-VP), (**b**) initiator:monomer weight ratio of P(2-VP), and (**c**) monomer type on pyridine-based polymer conversion using RS1.

An important parameter in the polymer synthesis is the temperature which could influence the reaction and the qualities of polymers (molecular weight and its molecular weight distribution) [42]. To investigate this effect, three temperatures (55, 65, and 75 °C) were considered for the 2-VP monomer conversion percentage using an initiator:monomer weight ratio at 4%wt in RS1 polymerizations, Figure 1a. It can be seen, in Figure 1a, at 2 h of reaction time, that the lowest temperature slows down the conversion. This observation is due to the fact that when the temperature is low, it reduces the thermal decomposition of BPO and produces a lower number of free radicals which react with the 2-VP [43]. Furthermore, the increase in the reaction temperature favored two effects: (a) the molecular diffusion and (b) the collision between 2-VP molecules and the free radicals [42]. The monomer conversion reaches a maximum value at 4 h and 2 h of reaction time for 65 °C and 75 °C, respectively. After that, the monomer conversion remained unchanged. Owing to the increase in the length of polymer chains, the viscosity of the reaction medium increases too as the polymerization advances to a point at which the motion of radicals slows down, thus affecting the monomer reaction rate. In consequence, the probability of collision between a monomeric molecule and a free radical is limited, and this effect is known as the glass effect [43]. For this reason, the maximum conversions were 62% and 55% at 6 h for 65 and 75 °C, respectively; these conversion percentages remained almost constant even if the polymerization time was increased to 24 h. It is worth noting that at 55 °C, the polymerization rate proceeded slower in comparison with other temperatures (65 and 75 °C) since it required 24 h to obtain 78% of conversion. In this case, the chains of polymer are more orderly in the solvent, which minimizes the glass effect and the collision between the species is more probable. Therefore, in this study 55 °C and 65 °C were selected as the temperatures for subsequent polymerizations of 2-VP and 4-VP, respectively, in the RS2. Meanwhile, the other experiments of initiator:monomer weight ratio and type of monomer in the RS1 were carried out at 65 °C.

Figure 1b shows the effect of the initiator:monomer weight ratio and the effect of the reaction time on the conversion of the monomer. As expected, the monomer conversion increased with the initiator:monomer weight ratio. It is well known that a higher initiator:monomer weight ratio produces more radical species which favors the polymerization reaction, hence, the conversion is increased [43,44]. When the initiator:monomer weight ratio diminished to half, the conversion also decreased around 20% at 6 h. Even with the reaction time increased to 24 h, the conversion percentage remained unchanged, Figure 1b. For this reason, the initiator:monomer weight ratio was established at 4% for subsequent polymerizations.

Finally, this study considered two monomer types, 2-VP and 4-VP, with the aim of determining the influence of their chemical structure in the polymerization using RS1. Figure 1c shows the monomer conversion versus polymerization. The maximum value of conversion was 62% for 2-VP at 6 h in contrast to 4-VP which achieved 76% at the same time. To determine the maximum conversion of the monomers, the polymerization time was increased to 24 h and, at such time, for 2-VP the conversion was almost equal to that reached at 6 h, meanwhile, for 4-VP 92% of conversion was obtained, Figure 1c. In the case of 4-VP (value not shown in Figure 1c), the polymerization time was extended to 48 h and the conversion increased approximately 6% in comparison to 24 h. For this reason, the selected time for subsequent polymerization was 24 h. It is worth mentioning that the difference between 2-VP and 4-VP conversion could be due to their chemical structures, since 4-VP monomer has less steric hindrance by the para-position of nitrogen in the aromatic ring which favors the polymerization. 

The results of solid content and monomer conversion are summarized in Table 1 for the two reaction systems. 

It is important to mention that the experiments carried out in the RS1 allowed the establishment of the conditions to synthesize the P(2-VP) at 55 °C and P(4-VP) at 65 °C in the RS2, both at 4%w initiator:monomer and 24 h of polymerization time. The RS2 increased the monomer conversion by approximately 27% and 14% with respect to RS1 for 2-VP and 4-VP, respectively. These results were mainly attributed to the change in the agitation system (magnetic to mechanical). During the polymerization, the viscosity of the medium reaction increased, which could interfere with the magnetic agitation and, as a result, the collision between the radicals and monomer decreased, obtaining lower conversions. The solid content was also affected by this effect, obtaining values lower than the theoretical one (30%w) for RS1. The reaction system selected for future polymerizations was the second (RS2) due to the increase in conversion and solids content; therefore, the elaboration of films and the characterization of polymers corresponds only to RS2. 

### 3.2. Film Characterization Results 

#### 3.2.1. FTIR and Micrograph Analysis of PVP 

**Figure 2:**
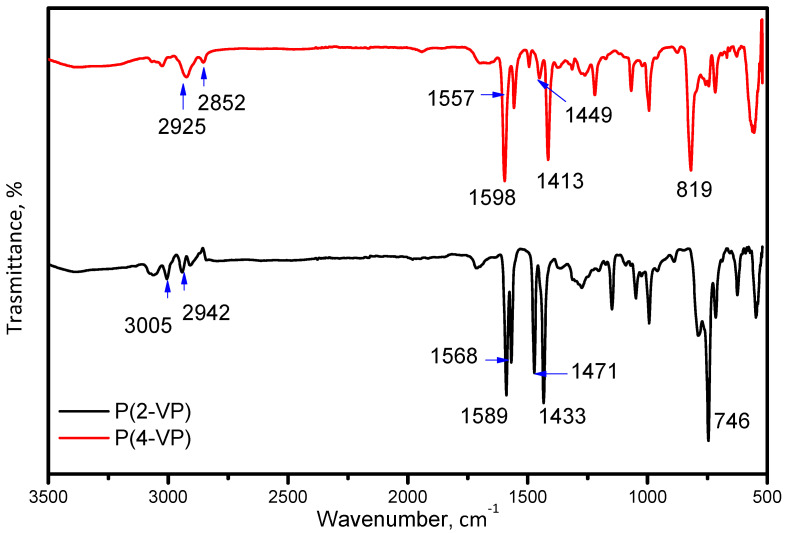
FTIR spectra of P(2-VP) and P(4-VP) using the RS2.

FTIR spectroscopy, optical microscopy, and SEM were used to characterize the P(2-VP) and P(4-VP). The FTIR spectra of PVP are shown in Figure 2. The observed peaks in the 2850–2952 cm^−1^ region are attributed to C–H stretching vibrations for both polymers while that at 3005 cm^−1^ in the P(2-VP) spectrum is due to =C-H symmetric stretching vibration. The C = N absorption peaks are assigned at 1598 cm^−1^ and 1557 cm^−1^ for P(4-VP) while those at 1589 cm^−1^ and 1568 cm^−1^ for P(2-VP) are due to the stretching vibration of the pyridine ring [45]. Moreover, the absorption bands at 1449 cm^−1^ and 1413 cm^−1^ for P(4-VP), as in the case of P(2-VP) at 1471 cm^−1^ and 1433 cm^−1^, appeared as result of the ring stretching vibration C = C. Finally, absorption bands at 819 cm^−1^ and 746 cm^−1^ for P(4-VP) and P(2-VP) were attributed to out-of-plane ring C–H bending and that around 1068 cm^−1^ can be assigned to the in-plane C–H bending [38,46]. 

Optical microscopy has been used to observe the different features of PVP such as the uniformity and the morphology among other characteristics. Figure S4a,b show optical images of P(2-VP) and P(4-VP) solution at 10% using the RS2, respectively. In general, the PVP film shows certain homogeneity on the substrate for both polymers. In order to characterize the morphology of PVP films obtained by RS2, these were also analyzed by metallographic microscope before (Figure S5a,b) and after (Figure S5c,d) the removal process of the residual monomer. The images of PVP films show a homogeneous surface before their treatment with water (Figure S5a,b), which agree with the optical image results (Figure S4a,b). However, some bubbles were generated due to the method of film preparation, and in the case of P(4-VP) their presence is more evident, Figure S5b. After the removal of monomers and/or oligomers, some cavities appeared (Figure S5c,d), this result is due to the swell of the polymer itself due to the contact with water. According to Mohd et al. [47], the presence of cavities, hollows, and pores in the films improved their hydrophilic properties as well as the adsorption of oxygen, water, or pollutants which could favor the photocatalytic activity of polymer films. 

**Figure 3:**
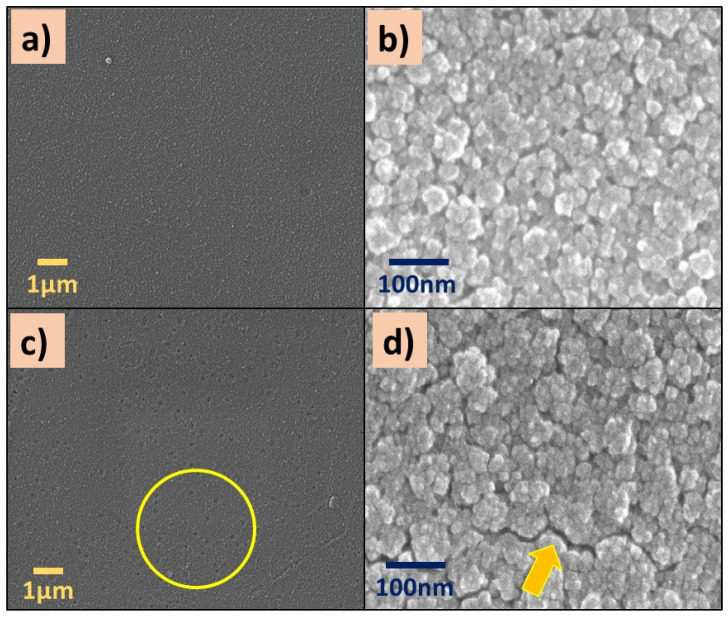
SEM images of (**a**,**b**) P(2-VP) and (**c**,**d**) P(4-VP) films using the RS2. SEM conditions: (**a**,**c**) 10 kV, LED, 10,000× magnification; (**b**,**d**) 5 kV, UED, 200,000× magnification.

SEM images of P(2-VP) and P(4-VP) films are displayed in Figure 3. Both polymers showed a rough surface (Figure 3a,c), and it becomes more evident as the magnification of the micrographs increases (from 10,000X to 200,000X). An irregular surface with some nanometer-sized aggregates and clusters can be seen in Figure 3b,d. In the case of P(4-VP), the formations of hollows (circle) and cracks (arrow) are visualized, in Figure 3d. The following circumstances can generate the defects on polymer films: (a) the substrate–polymer–solvent interaction, (b) the solvent evaporation method, and (c) the coating conditions (angular speed, application time, among others).

The presence of bubbles formed during the preparation of the polymer film generates holes by the solvent evaporation (IPA). Meanwhile, the cracks were generated when the IPA evaporation rate was fast enough, causing the polymer contraction and, in consequence, the polymer chains did not spread on the substrate. These results suggest that polymer aggregates and clusters are associated with the interaction among solvent, the substrate (glass), and the polymer solution. When the interaction between the solvent and the substrate dominates, a rough film is formed (Figure 3b,d) and it is not homogeneous. On the contrary, if the interaction between the polymer and substrate is favorable, homogeneous films are formed. Similar results were obtained by Juey H. Lai [48] who studied the deposition of polymers film (PS-PMMA) using different solvents by spin coating. They explained that the formation of aggregates on the film is due to the fast evaporation of the low-volatility solvent.

#### 3.2.2. Composite Characterization Results

**Figure 4:**
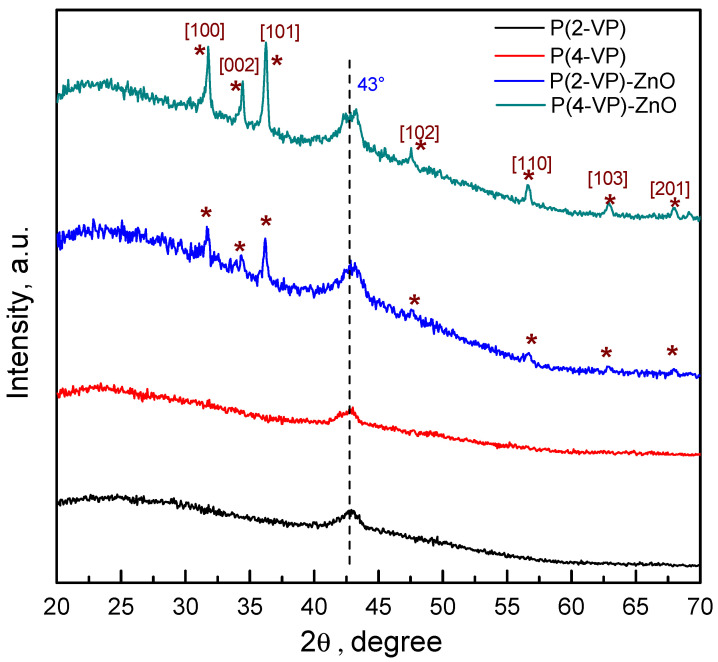
GIXRD patterns of PVP and PVP-ZnO films obtained by spin coating.

To verify the crystalline and the chemical structure of the films, the GIXRD patterns were obtained and results are shown in Figure 4. For pure PVP, films exhibit a small peak at 43° which indicated that it has a semicrystalline nature. In the case of composites, both PVPs show the characteristic diffraction of ZnO, indicating that the synthesis method leads to successful film formation without modifying the crystalline structure of ZnO. The series of peaks can be indexed to the wurtzite structure of ZnO (JCPDS card #98-016-6243). Nevertheless, in the diffraction patterns of the PVP-TiO_2_ composites, the series of peaks attributable to TiO_2_ phase were not possible to identify, which may be due to the existence of uncoated zones of semiconductor or its lower amount in composite, Figure S6. 

Optical microscopy was used to discern the dispersion of the metal oxide particles on the polymer, also known as the features, such as morphology and uniformity of the film. Furthermore, it allows identification of large agglomerates and distinguishing very dense materials (metal oxide) from less dense materials (polymers), and the information may come from a few tens or hundreds of micrometers below the surface of the sample. Figure S7 shows the images in the bright (Figure S7a,c,e,g) and dark field (Figure S7b,d,f,h) of composites. According to the image of P(2-VP)-TiO_2_, the semiconductor formed several channels (white zones, Figure S7b) on film. 

Comparing P(2-VP)-TiO_2_ against P(4-VP)-TiO_2_, it is observed that there is higher retention of SC in P(4-VP) than in P(2-VP)-TiO_2_, because the dark field image shows major uniformity of SC on polymer (Figure S7b,f) and the cavities of the polymer are more evident. In the case of P(2-VP)-ZnO, some air bubbles can be observed due to the preparation of films (Figure S7c,d) and, finally, for P(4-VP)-ZnO, the presence of some aggregates is possible to identify (Figure S7g,h). 

**Figure 5:**
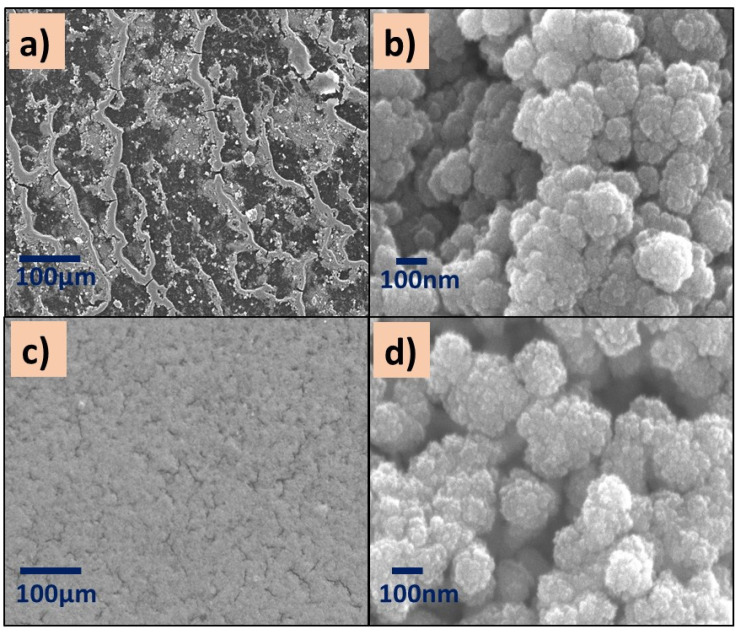
SEM images of (**a**,**b**) P(2-VP)-TiO_2_ and (**c**,**d**) P(2-VP)-ZnO. SEM conditions: (**a**,**c**) 10 kV, LED, 200× magnification; (**b**,**d**) 5 kV, UED, 100,000× magnification.

With the aim to enrich the results of optical microscopy, the morphology of composites was characterized by SEM as shown in Figure 5. Generally, SEM is used to obtain surface information from small regions at higher magnifications and, in addition, to acquire elemental microanalysis of specific regions. 

All images showed aggregates and cluster particles independently of the kind of polymer, which could provide active sites for the pollutant. Specifically, the surface of P(2-VP)-TiO_2_ films at low SEM amplification (200×) shows an irregular surface with a channel-like morphology (Figure 5a) in addition to the existence of SC agglomerations over the channels. These images agree with the optical microscopy images. This is in contrast to P(2-VP)-ZnO which presents a more homogeneous surface since the ZnO is in the clusters of the polymer film (Figure 5c,d). Even when the reported sized of commercial TiO_2_ (21 nm) is smaller than the ZnO (100 nm), the ZnO seems to be uniformly distributed over the surface of P(2-VP), indicating that the interaction between polymer and SC is better in comparison to TiO_2_. 

**Figure 6:**
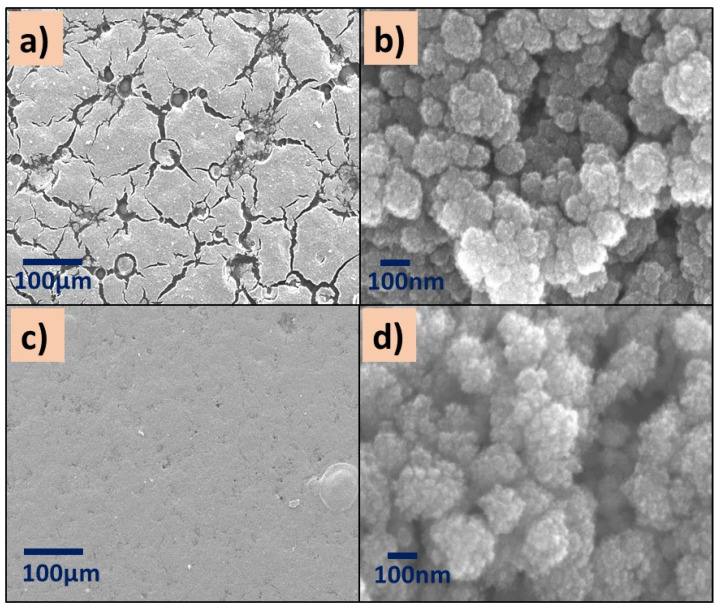
SEM images of (**a**,**b**) P(4-VP)-TiO_2_ and (**c**,**d**) P(4-VP)-ZnO. SEM conditions: (**a**,**c**) 10 kV, LED, 200× magnification; (**b**,**d**) 5 kV, UED, 100,000× magnification.

The micrographs of P(4-VP)-TiO_2_ exhibit surface defects such as cracks (Figure 6a,b), while the distribution of ZnO nanoparticles on the P(4-VP) film is uniform, Figure 6c,d. In summary, the presence of cluster particles in all composites is clear, suggesting that the interaction between water and PVP dominates during the removal of unreacted monomer, in comparison with the SC–PVP interaction. This effect is possible to observe in the following order in the films: P(2-VP)-TiO_2_ > P(4-VP)-TiO_2_ > P(2-VP)-ZnO > P(4-VP)-ZnO.

**Figure 7:**
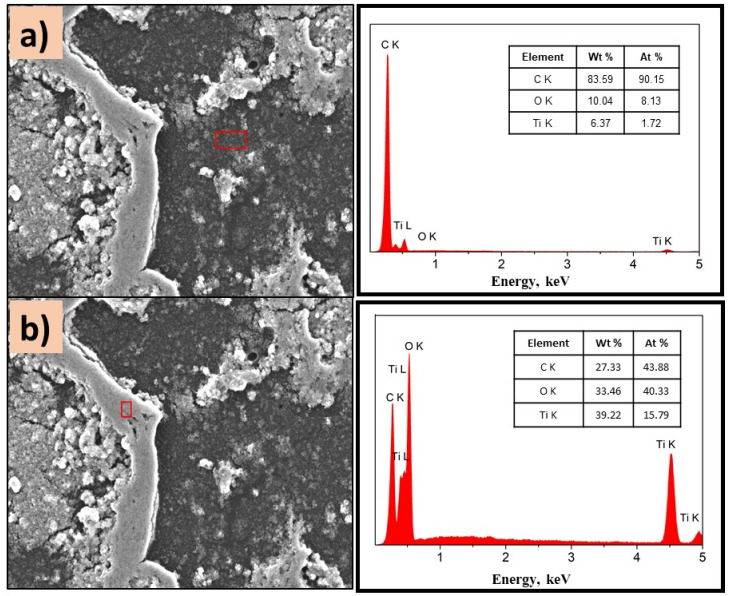
SEM images of P(2-VP)-TiO_2_ analyzed in (**a**) dark zone and (**b**) white zone. The inset figure corresponds to the elemental composition of the films determined by selected EDS area. Conditions: 10 kV, LED, 1000× magnification.

**Figure 8:**
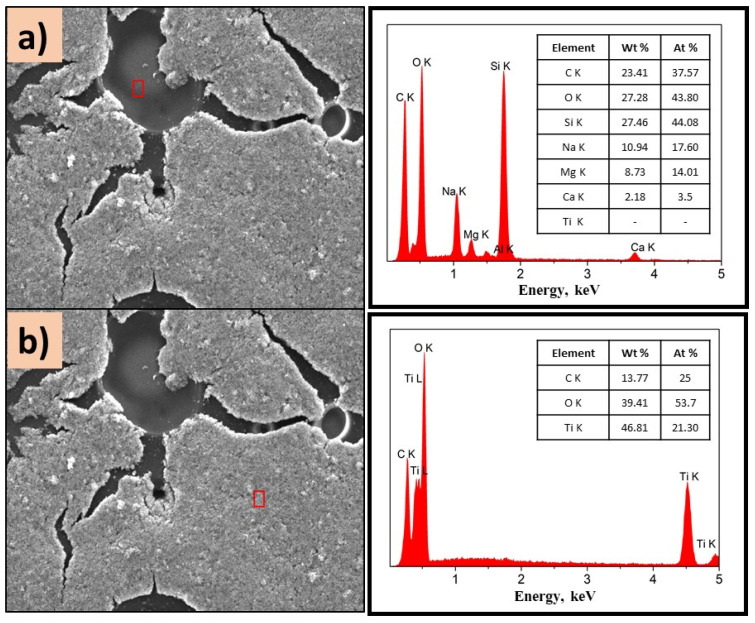
SEM images of P(4-VP)-TiO_2_ analyzed in (**a**) dark zone and (**b**) white zone. The inset figure corresponds to the elemental composition of the films determined by selected EDS area. Conditions: 10 kV, LED, 1000×.

To know the distribution of TiO_2_ in the composite, EDS analysis was performed on different areas of the films, Figure 7 and Figure 8. 

Figure 7a,b show the morphology of the P(2-PV)-TiO_2_ composite which presented dropping areas (dark zones) and rising areas (white zones) that provide channels of SC particles. Through EDS analysis of the lower areas observed in the image of P(2-VP)-TiO_2_ in Figure 7a, carbon was mainly detected due to PVP, as well as Ti and O from TiO_2_. In the case of dark zones of P(4-VP)-TiO_2_, EDS analysis also identified Na, Si, Mg, and Al as belonging to the film substrate (glass), Figure 8a. For the white zones, the existence of Ti and O confirms the presence of cluster TiO_2_ on the film, Figure 7b and Figure 8b. SEM images of PVP-TiO_2_ films corroborate the irregularities on these surfaces, consistent with the results obtained in optical microscopy. 

**Figure 9:**
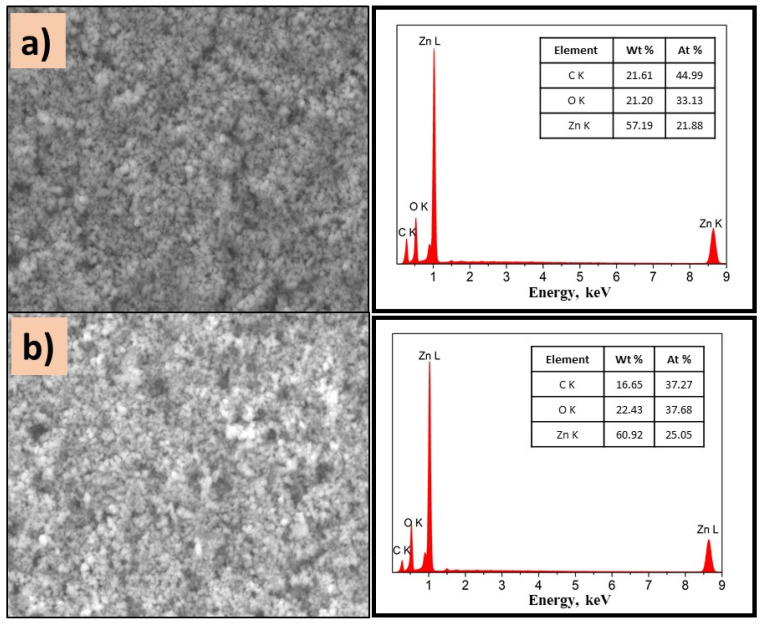
SEM images of (**a**) P(2-VP)-ZnO and (**b**) P(4-VP)-ZnO; the inset images are the corresponding EDS spectra. Conditions: 10 kV, LED, 10,000× magnification.

On the other hand, ZnO composites exhibit homogeneous surfaces independently of PVP. As displayed in Figure 9a,b, the presence of Zn, O, and C is detected, which confirms the existence of well-dispersed ZnO on the surface of the film. It is important to mention that the semiconductor type influenced the morphology of composites, due to the intermolecular forces. 

**Figure 10:**
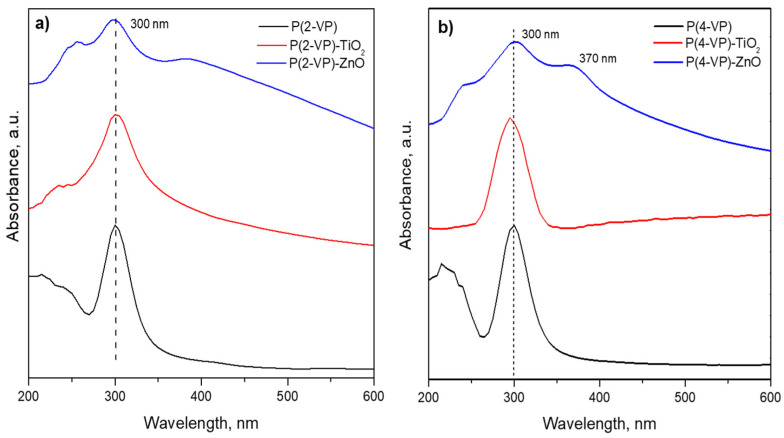
UV–Vis spectra of (**a**) P(2-VP) composites and (**b**) P(4-VP) composites.

To determine the optical properties of prepared composites, the optical bandgap energies were calculated using a Tauc plot derived from the UV–Vis spectra, Figure 10. The peak appearing around 300 nm was related to the absorption of pyridine and benzene groups in PVP films [49,50]. These signal remains in the composite films due to the absorption of TiO_2_ and ZnO in the same region [20]. However, P(4-VP)-ZnO also shows an additional signal at 370 nm that could be attributed to the presence of additional energy levels [51].

The bandgaps of P(2-VP) and P(4-VP) were 3.73 and 3.8 eV, respectively, and these values indicated that the absorption of PVP is mainly within ultraviolet light. In the case of composites, the bandgap was 3.5, 3.2, 3.8, and 2.8 eV for (P-2VP)-TiO_2_, (P-2VP)-ZnO, (P-4VP)-TiO_2_, and (P-4VP)-ZnO. The above results indicated that the presence of semiconductors reduces the bandgap of composites slightly, increasing the capacity of absorption of UVA. In particular, the case of P(4-VP)-ZnO exhibited a relatively low absorption capacity in the visible light, which would indicate an increase in its photocatalytic activity, as will be shown later. 

### 3.3. Photocatalytic Activity of PVP and Composites 

**Figure 11:**
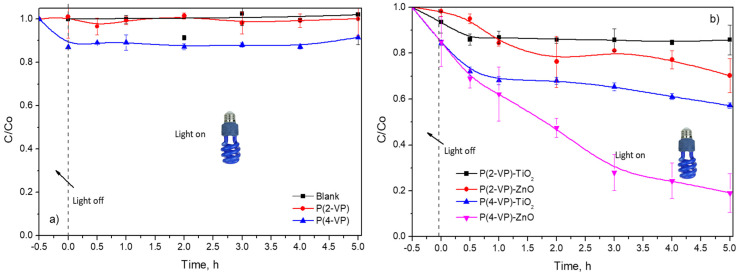
Dimensionless concentration profiles of MO (10 mg L^−1^) in presence of (**a**) PVP and (**b**) composites by photocatalysis.

Photocatalytic activity of the prepared films was evaluated for the degradation of MO and BA under ultraviolet light at 365 nm. Before the photocatalytic evaluation, the reactor remained stirred in the presence of PVP or composites under dark conditions for 30 min, to carry out the adsorption of organic compounds on the surface of films. As displayed in Figure 11a, no color removal of MO was observed during 5 h of irradiation without catalysts (blank test). In the presence of the PVP, low contribution of MO color removal via adsorption process was obtained (Figure 11a), therefore, the irradiation source was required to activate the materials. Nevertheless, both PVPs exhibit negligible MO photodegradation during 5 h (around 10%).

In the case of composites, P(2-VP)-TiO_2_ and P(2-VP)-ZnO only achieve 14% and 31% of MO degradation, respectively, during 5 h, while P(4-VP)-ZnO presented a higher dye removal of around 81% during the same time. The photocatalytic activity increased according to the following order: P(2-VP)-TiO_2_ < P(2-VP)-ZnO < P(4-VP)-TiO_2_ < P(4-VP)-ZnO. As expected, the P(4-VP)-ZnO film showed higher photocatalytic activity, since the composite had better dispersion of ZnO on polymer and presented a lower value of the bandgap. For this reason, thereafter, P(4-VP)-ZnO composite was employed for subsequent experiments.

It is worth mentioning that composites with P(4-VP) showed higher MO removal than P(2-VP) and between the metallic oxides, ZnO was better that TiO_2_, indicating that the type of semiconductor and polymer had an influence on photocatalytic activity of composites in the dye degradation. For the polymer matrix, it is possible that the chemical structure of P(2-VP) affects the interaction of free electrons found in the pyridine ring and the semiconductor (ZnO and TiO_2_) due to the heteroatom position. This interaction between PVP and semiconductor can involve hydrogen bonds, favoring the charge conduction. 

It is essential to mention that P(4-VP)-ZnO catalyst has not been used for the elimination of MO (as displayed in Table 2), however, this catalyst is a viable option to remove organic compounds. 

**Figure 12:**
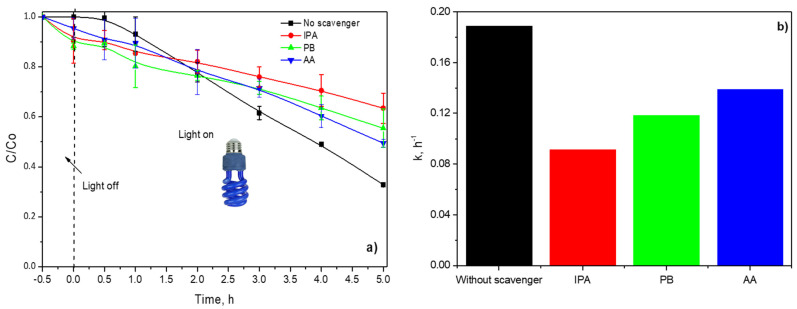
(**a**) Dimensionless concentration profiles of BA in presence of active species trapping and (**b**) calculated pseudo-first-order constants.

The BA photodegradation was also studied with and without active species trapping (scavenging), as displayed in Figure 12. The presence of IPA inhibited around 50% of the degradation rate of BA, indicating that •OH were dominant reactive species photogenerated in the reaction system, Figure 12a. 

P-Benzoquinone (PB) and ascorbic acid (AA) were used to scavenge the O_2_•^−^ and h^+^, respectively. Both scavengers diminished approximately 20% and 15% of the dimensionless concentration of BA in comparison with blank, Figure 12b. Consequently, O_2_•^−^ and h^+^ also take part in BA photodegradation during the irradiation of P(4-VP)-ZnO with UVA. 

The experimental data were fitted to a pseudo-first-order model; the kinetic constants are shown in Figure 12b. As expected, the lower value of the kinetic constant is with IPA, indicating that •OH was mainly responsible for BA photodegradation and color removal of MO dye.

## 4. Conclusions

The P(2-VP)-TiO_2_, P(2-VP)-ZnO, P(4-VP)-TiO_2_, and P(4-VP)-ZnO composites have been prepared by spin coating method on glass substrates. P(2-VP) and P(4-VP) were synthetized by polymerization in solution, demonstrating that the reaction temperature and initiatior:monomer weight ratio influenced the conversion grade, as well as the stirring mode. The amine position in the hetero-ring has an effect on polymerization, being the best option to polymerize the 4-VP.

The GIXRD analysis indicated that the PVP has a semicrystalline nature due to the presence of a peak around 43°. The morphology studies (SEM) showed the presence of aggregates and clusters of particles for all composites, however, the P(4-VP)-ZnO and P(4-VP)-TiO_2_ composites revealed a better homogeneity on film which indicated good interaction between polymer and semiconductor. 

The composites presented different activity in the MO photodegradation. A higher photocatalytic activity was obtained with P(4-VP)-ZnO achieving 80% of MO degradation during 5 h of irradiation, which is indicative of an efficient separation of photogenerated species after their excitation with a UVA lamp. Furthermore, the formation of •OH together with the photogenerated holes and O_2_•^−^ participate in BA photodegradation. Finally, it should be noted that these composites were efficiently employed in photocatalytic application for organic compound degradation for the first time. 

## Figures and Tables

**Table 1:** Solid content and monomer conversion (%) for both reaction systems.

Monomer	2-VP		4-VP	
Reaction temperature	55 °C		65 °C	
Reaction system	RS1	RS2	RS1	RS2
Solids, % w	23.4	28.2	26.4	30
Conversion, % 24 h	64	91	92	100

**Table 2:** Comparison of MO elimination percentages reported in the literature using diverse photocatalysts.

Photocatalyst (Synthesis Method)	Experimental Condition	Irradiation Source	% Pollutant Elimination	Refs.
P(4-VP)-ZnO (Spin coating method)	[MO] = 10 mg L^−1^	UVA lamp (365 nm, 10 W)	81% of dye removal within 5 h	Present study
ZnO-Ag based polymer composites film (Chemical precipitation method)	[MO] = 5 × 10^−5^ M m_cat_ = 1 g	Xe lamp at 437 nm (4.9 mW cm^−2^)	95 degradation efficiency% after 250 min of irradiation	[52]
Ag/MoO_3_/TiO_2_ nanocomposite film (Sol–gel method)	[MO] = 10 mg L^−1^ pH = 3.0 m_cat_ = 12 g	5 UV lights of 20 W	96.5% of MO was degraded under 5.5 h UV irradiation	[53]
Porous polymer supported Ag-TiO_2_ (In situ solvothermal process)	[MO] = 100 mg L^−1^ [cat] = 50 g L^−1^	Xenon lamp (100 mW cm^−2^) at 200–2500 nm (without filter)	81.4% of degradation at 3 hr	[54]
TiO_2_/Cs-Mt CS: chitosan Mt: montmorillonite (layer by layer)	[MO] = 20 mg L^−1^ pH = 6.5	45 W fluorescent lamp	98.7% of MO removal	[55]
TiO_2_/Fe-PPG PPG: PANI-polyvinyl alcohol-glutaraldehyde (layer by layer)	[MO] = 20 mg L^−1^ pH = 6.8	45 W fluorescent lamp	95.4% of MO degradation after 1 h of irradiation	[56]
Cu@PCNF PCNF: porous carbon nanosphere film (Ultrasonic spray pyrolysis)	0.6 wt% MO solution	Sunlight (20,000 ± 2000 lux)	The degradation rate reached 80% after 4 h	[57]

## Data Availability

Data sharing not applicable.

## Supplementary Materials

The following supporting information can be downloaded at: https://www.mdpi.com/article/10.3390/polym14214666/s1, Figure S1: A schematic illustration of the first reaction system (SR1) to synthesis of P(2-VP) and P(4-VP), Figure S2: A schematic illustration of the second reaction system (SR2) to synthesis of P(2-VP) and P(4-VP), Figure S3: A schematic illustration of the preparation of PVP-metal oxides composites, Figure S4: Optical microscope images of (a) P(2-VP) and (b) P(4-VP) solution at 10%v at 4X magnification, Figure S5: Metallographic microscope image of (a,c) P(2-VP) and (b,d) P(4-VP) solution at 10%v (a,b) before and (c,d) after the monomer residual removal using the RS2, Figure S6: XRD pattern of PVP-TiO2 films obtained by spin coating, Figure S7: Metallographic microscope image of (a,b) P(2-VP)-TiO2, (c,d) P(2-VP)-ZnO, (e,f) P(4-VP)-TiO2, (g,h) P(4-VP)-ZnO after the residual monomer removal at 10X magnification.
