# Supplementary material for: Synthesis and Characterization of Poly(2-vinylpyridine) and Poly(4-vinylpyridine) with Metal Oxide (TiO2, ZnO) Films for the Photocatalytic Degradation of Methyl Orange and Benzoic Acid
Source: Polymers (Basel). 2022 Nov 1;14(21):4666. doi: 10.3390/polym14214666 (PMC9657262; doi:10.3390/polym14214666)
Supplement: Supplementary file 1 [file polymers-14-04666-s001.zip › polymers-1935890-supplementary.pdf]

Supporting information

Synthesis and characterization of poly(2-vinylpyridine) and poly(4-vinylpyridine) with metal oxide (TiO_2, ZnO) films for the photocatalytic degradation of methyl orange and benzoic acid

Iririana Martínez[1], Ricardo Santillán*[2], Iliana Fuentes[1], Julia L. Rodríguez S.*[1], J. Alberto-Andraca Adame[3], Hugo Martínez Gutiérrez[4]

[1]Lab. Ing. Química Ambiental. ESIQIE–Instituto Politécnico Nacional.
Zacatenco, C.P. 07738 México, D.F. México

[2]Lab. de Polímeros, ESIQIE-Instituto Politécnico Nacional,
Zacatenco, México, 07738, CDMX.

[3]Departamento de Ciencias Básicas, UPIIH—Instituto Politécnico Nacional,
Hidalgo, 42050, México

[4]Centro de Nanociencias y Micro y Nanotecnologías, Instituto Politécnico Nacional, Ciudad de México, México.

Corresponding authors:

*E-mail: rsantillanp@ipn.mx (Ricardo Santillán)
jlrodriguezs@ipn.mx (Julia L. Rodríguez)

Materials and methods

Figure S1 shows the main steps carried out during the synthesis of P(2-VP) and P(4-VP).

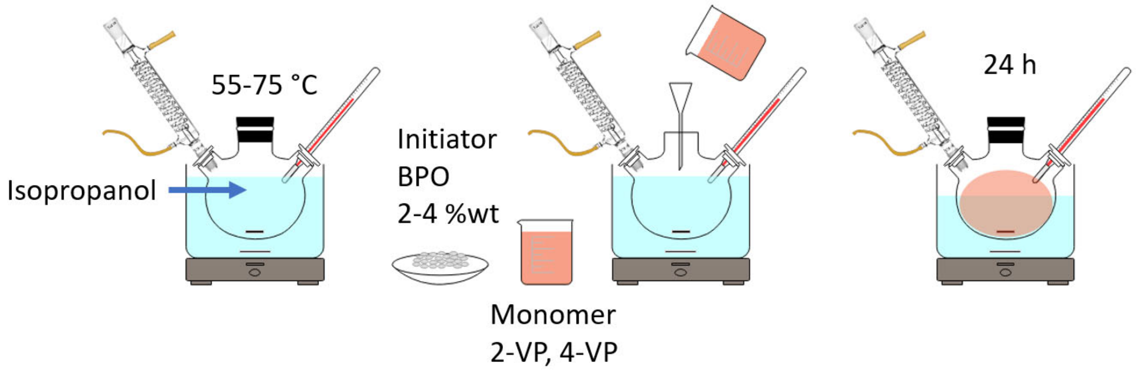

Figure S1. A schematic illustration of the first reaction system (SR1) to synthesis of P(2-VP) and P(4-VP).

The agitation system was changed in the second reaction system in comparison with SR1 as shown in Figure S2.

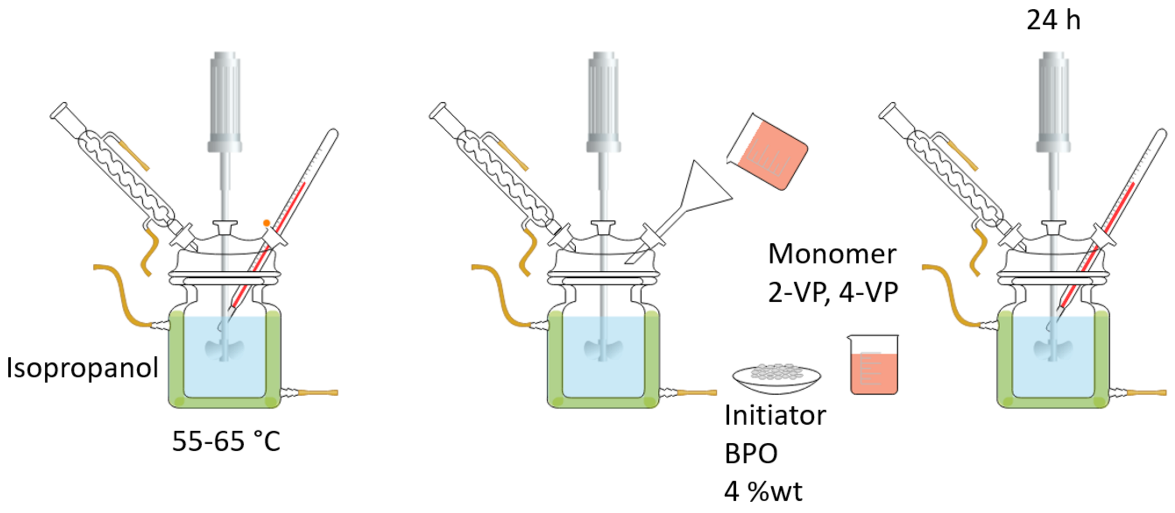

Figure S2. A schematic illustration of the second reaction system (SR2) to synthesis of P(2-VP) and P(4-VP).

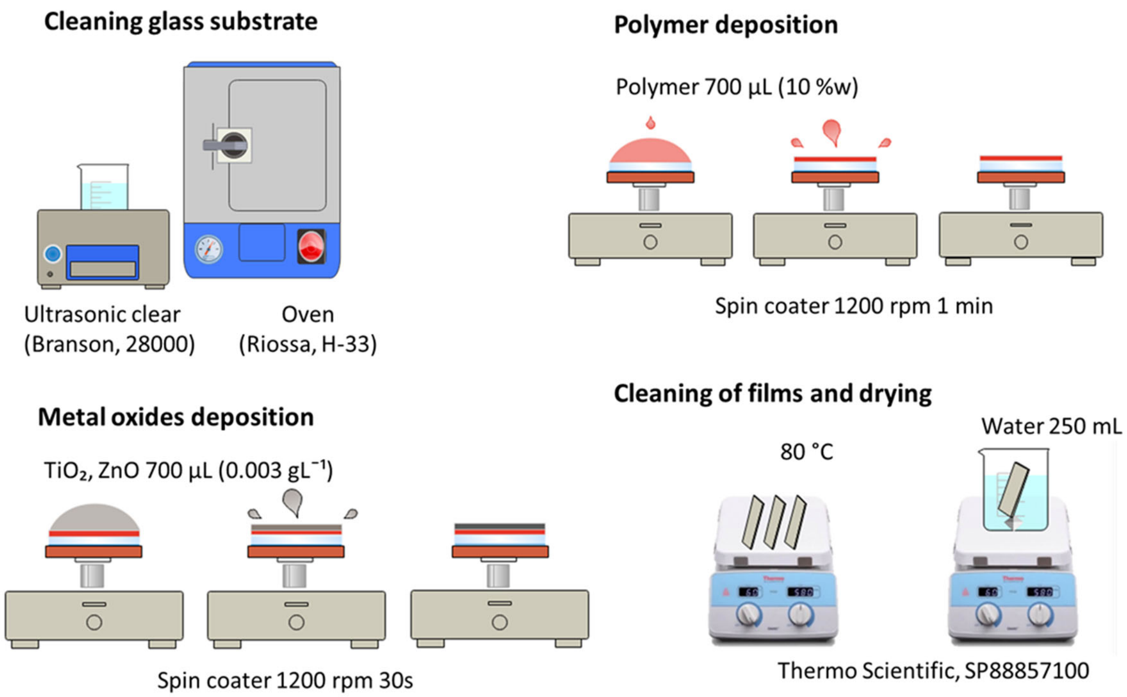

Figure S3. A schematic illustration of the preparation of PVP-metal oxides composites.

Results

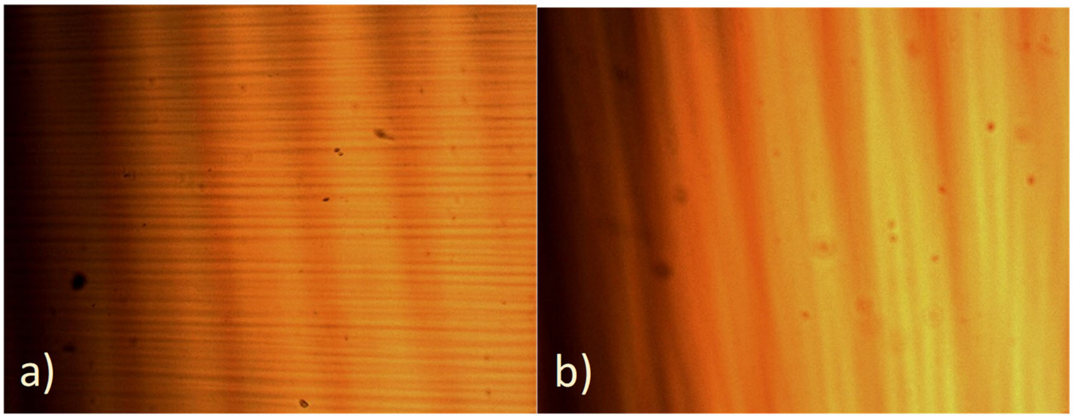

Figure S4. Optical microscope images of a) P(2-VP) and b) P(4-VP) solution at 10%v at 4X magnification.

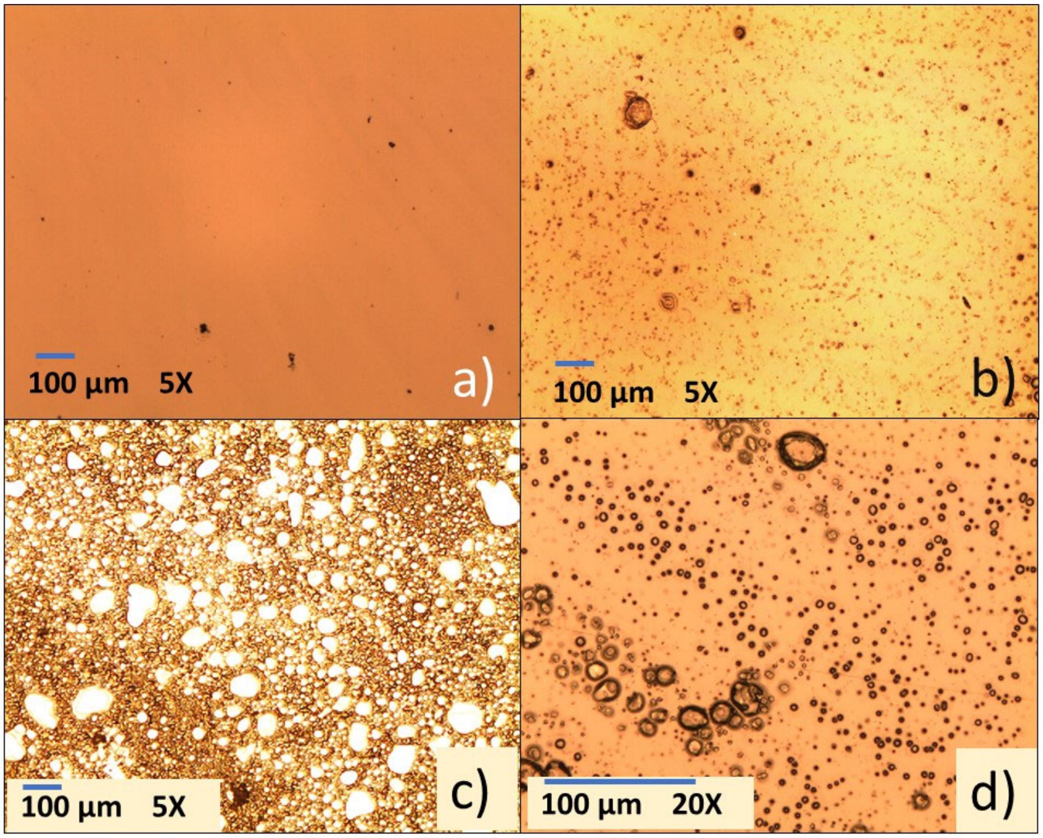

Figure S5. Metallographic microscope image of (a, c) P(2-VP) and (b, d) P(4-VP) solution at 10%v (a, b) before and (c, d) after the monomer residual removal using the RS2.

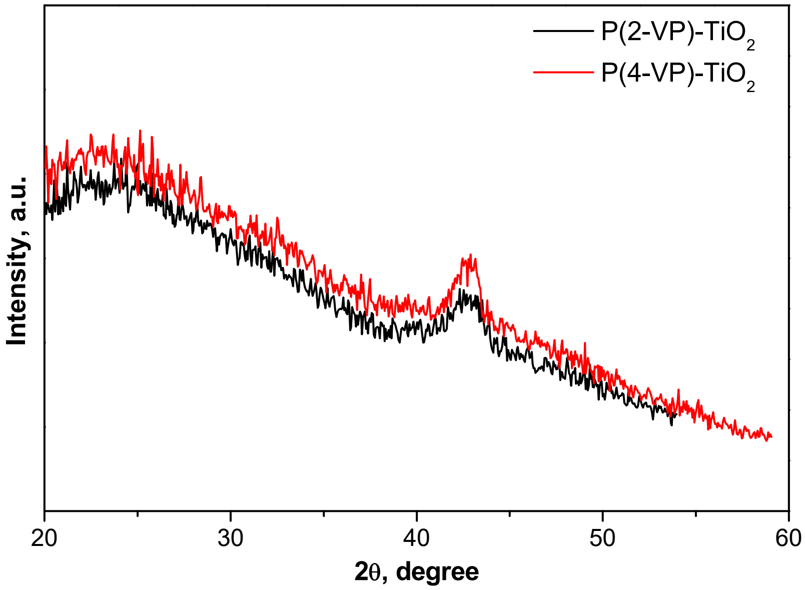

Figure S6. XRD pattern of PVP-TiO2 films obtained by spin coating.

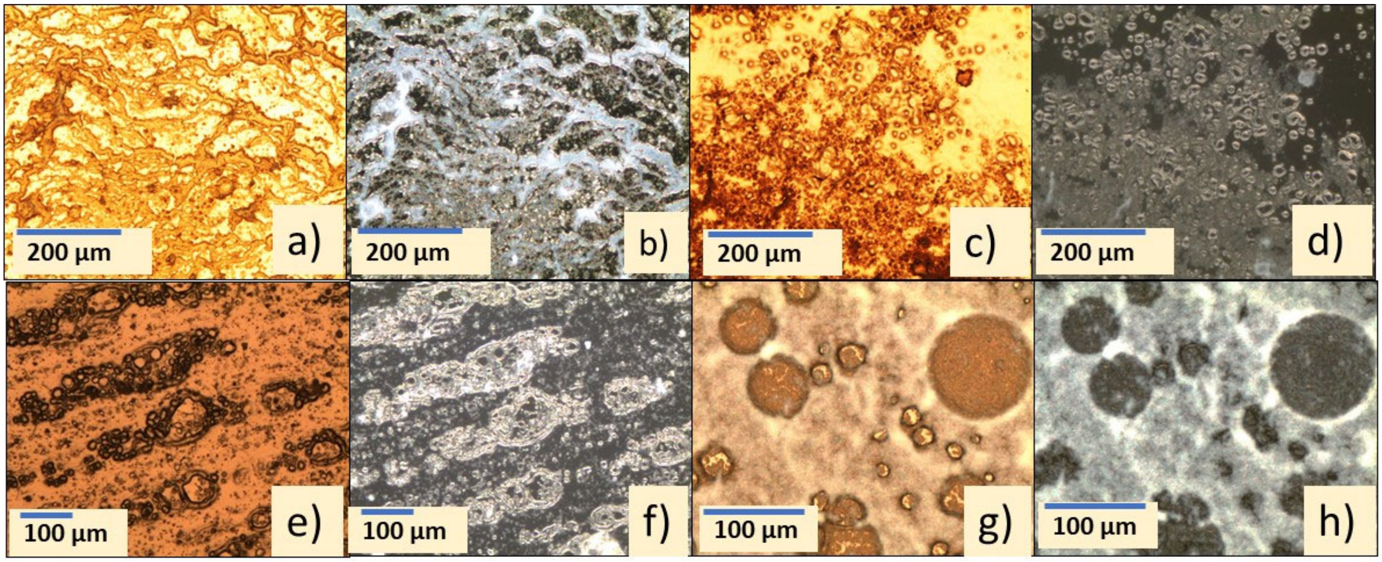

Figure S7. Metallographic microscope image of (a ,b) P(2-VP)-TiO_2, (c, d) P(2-VP)-ZnO, (e ,f) P(4-VP)-TiO_2, (g, h) P(4-VP)-ZnO after the residual monomer removal at 10X magnification.
